# Causal Relationships Between Osteoarthritis and Senile Central Nerve System Dysfunction: A Bidirectional Two-Sample Mendelian Randomization Study

**DOI:** 10.3389/fnagi.2021.793023

**Published:** 2022-03-04

**Authors:** Yuanqing Cai, Guangyang Zhang, Jialin Liang, Zhaopu Jing, Rupeng Zhang, Leifeng Lv, Xiaoqian Dang

**Affiliations:** Department of Orthopedics, The Second Affiliated Hospital of Xi’an Jiaotong University, Xi’an, China

**Keywords:** Parkinson’s disease, Alzheimer’s disease, ischemic stroke, osteoarthritis, Mendelian randomization

## Abstract

**Background:**

The relationship between osteoarthritis (OA) and senile central nervous system dysfunctions (CNSDs), including Parkinson’s disease (PD), Alzheimer’s disease (AD), and ischemic stroke (IS) has gradually attracted attention. At present, the causal relationship between OA and CNSD remains unclear. The aim of this study was to assess the causal effects of CNSD and OA using Mendelian randomization (MR).

**Methods:**

Genome-wide association study summary data for CNSD and OA were obtained. Single-nucleotide polymorphisms (SNPs) were selected as instrumental variables (IVs), and significant (*P* < 5.0 × 10^–8^) and independent (*r*^2^ < 0.1) SNPs were extracted for bidirectional MR analysis. Inverse variance weighted (IVW) was used to assess these causal relationships. The results are reported as odds ratios (ORs). Subsequently, heterogeneity was tested using the Cochran’s *Q* test, pleiotropy was tested using the MR-Egger intercept, and sensitivity analysis was performed using the leave-one-out sensitivity test.

**Results:**

The MR results of the causal relationship between PD and OA showed that there was a positive causal effect of OA on PD, which was estimated by IVW (OR = 1.194, 95%CI = 1.036, 1.378; *P* = 0.0144). Moreover, the MR analysis by IVW also showed that IS had a positive effect on OA (OR = 1.033, 95%CI = 1.002, 1.066; *P* = 0.0355). These results are reliable and stable, as confirmed by sensitivity tests.

**Conclusion:**

This study showed a positive causal effect of OA on PD, but there was a null effect of OA on AD and OA on IS.

## Introduction

Parkinson’s disease (PD), Alzheimer’s disease (AD), and ischemic stroke (IS) are the three most common central nervous system dysfunctions (CNSDs) in elderly people. Paralysis agitans (PD) is a neurodegenerative disease characterized by slow movement, static tremor, and increased muscle tension (Hayes, 2019). The main pathological change in PD is the degeneration and death of dopaminergic neurons in the substantia nigra of the midbrain, which leads to a significant decrease in dopamine content in the striatum (Dickson, 2018). Alzheimer’s disease, also known as senile dementia, is a progressive neurodegenerative disease with hidden onset and is characterized by memory disorder, aphasia, apraxia, agnosia, executive dysfunction, and personality and behavior changes, and its etiology is yet to be fully elucidated (Lane et al., 2018; Weller and Budson, 2018). The incidence of IS is growing because of the increase in obesity and decrease in exercise. IS refers to brain tissue necrosis caused by stenosis or occlusion of blood supply arteries (carotid and vertebral arteries), resulting in insufficient blood supply to the brain (Rabinstein, 2020). With the advent of an aging society, there has been a sharp increase in the number of patients suffering from senile CNSD. Previous studies have shown that the number of AD cases will increase to around 115–135 million by 2050 (Tamfu et al., 2021), and the incidence rates of PD are about 108–257/100,000 and 11–19/100,000 per year in Europe (Balestrino and Schapira, 2020). Thus, much more attention should be paid to senile CNSD, its complications, etiologies, etc.

Degenerative osteoarthropathy, also known as osteoarthritis (OA), is a degenerative disease. The pathogenesis of OA is degeneration and injury of the articular cartilage and reactive hyperplasia of the articular edge and subchondral bone (Geyer and Schönfeld, 2018). The clinical manifestations are pain, tenderness, stiffness, joint swelling, limited movement, and joint deformity (Xia et al., 2014; Vincent, 2019). Many patients undergo total joint arthroplasty for pain relief and joint function recovery (Robinson et al., 2018; Lu and Phillips, 2019). In the United States, 22.7 million activity limitations have been attributed to arthritis, especially to OA (Frieden et al., 2010), and OA also brings a large economic burden to society. Therefore, scholars have increasingly focused on OA. In recent years, there has been a growing attention to the relationship between OA and senile CNSD. For example, studies have suggested that OA-related inflammation and pain might increase the risk of PD and ADL (Ikram et al., 2019; Roper et al., 2020; Feng et al., 2021), and an increasing number of studies have demonstrated the causal effects of OA on stroke (Hsu et al., 2017; Yeh et al., 2021). However, most of these studies were retrospective, in which the confounders and biases, such as selection bias and memory bias, could not be eliminated, and reverse causality might also have occurred. Therefore, the causal associations between CNSD and OA have not yet been clearly established.

Mendelian randomization (MR) is a method mainly used in epidemiological etiological investigations in recent years (Xu et al., 2020; Qu et al., 2021). In MR studies, exposure is regarded as an intermediate phenotype, which is determined by genotype, and the differences of genotype [generally single-nucleotide polymorphisms (SNPs)] are used as instrumental variables (IV) to study the association effect between genotypes and diseases, so as to simulate the association between exposure and disease. Therefore, MR studies are not affected by the confounders of traditional epidemiological methods (such as retrospective studies) and reverse causality. Thus, MR analysis has been widely used in many studies (Luo et al., 2020; Nazarzadeh et al., 2020). To address this issue, this study applied bidirectional two-sample MR to evaluate the causal relationships between CNSDs and OA.

## Materials and Methods

### Study Design

**FIGURE 1:**
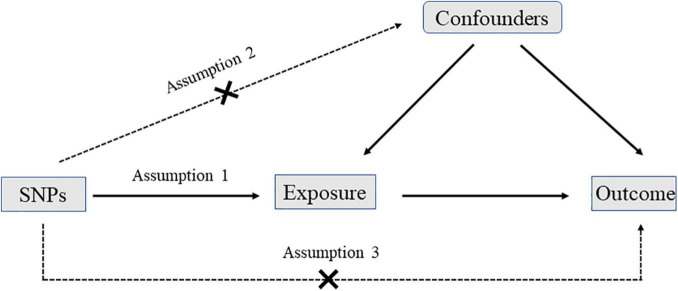
The diagram of Mendelian randomization (MR): three assumptions should be met: first, the single nucleotide polymorphisms (SNPs) should be strongly associated with exposures; second, the selected SNPs should be independent of confounders; third, the SNPs should affect outcomes only by exposures rather than via a direct correlation.

In the MR analysis, three assumptions should be met: first, the SNPs should be strongly associated with exposures; second, the selected SNPs should be independent of confounders; third, the SNPs should affect outcomes only by exposures rather than via a direct correlation (Figure 1).

In this study, SNPs were chosen as IVs to perform bidirectional two-sample MR to determine the causal relationship between CNSD and OA using genome-wide association study (GWAS) data. A bidirectional design was applied to assess the causal effects of CNSD on OA and to evaluate whether there are causal effects of OA on CNSD.

### Genome-Wide Association Study Summary Data of Central Nervous System Dysfunctions and Osteoarthritis

This two-sample MR analysis was performed based on the GWAS summary data. All summary data were from European ancestry. Genetic predictors of AD were obtained from a published GWAS meta-analysis, which was based on 17,008 AD cases and 37,154 controls (Lambert et al., 2013). Genetic predictors of PD were obtained from recently published data^1^ from the International Parkinson’s Disease Genomics Consortium, which was based on 33,674 PD cases and 449,056 controls. Genetic predictors of IS were also obtained from a published GWAS, which was based on 7,193 IS cases and 406,111 controls (Malik et al., 2018). The GWAS summary statistics of OA were obtained from published genome-wide analyses of UK Biobank data, which were based on 455,211 individuals of European-ancestry (77,052 OA cases and 378,169 controls) (Tachmazidou et al., 2019).

### Instrumental Variable Selection

To perform a two-sample MR analysis, SNPs were chosen as IVs. Genome-wide significant (*P* < 5 × 10^–8^) SNPs were extracted as IVs, and linkage disequilibrium was tested (*r*^2^ < 0.1); SNPs with linkage disequilibrium were excluded. Finally, SNPs with an *F*-value ≥ 10 were selected for further analysis.

### Statistical Analysis

The standard inverse variance weighted (IVW) method was used to analyze the causal relationships between CNSDs and OA. The Wald ratio of each SNP was calculated to assess the causal effects of each SNP on outcome, and the inverse variances of SNPs were used as weights for meta-analysis to evaluate the combined causal effect. Furthermore, MR Egger, weighted median, simple mode, and weighted mode were also used to evaluate the causal relationships between CNSDs and OA. IVW provides unbiased estimates, as long as all genetic variants are valid instruments. The weighted median provides valid estimates if at least 50% of the weight comes from valid variants. MR-Egger can have low statistical power, so we concentrated on the direction and effect size rather than statistical significance (Luo et al., 2020). MR analysis was first performed to assess the causal effect of CNSD on OA and then performed in the opposite direction.

Finally, heterogeneity was tested using Cochran’s *Q* test, pleiotropy was tested using MR-Egger intercept, and sensitivity analysis was performed using the leave-one-out sensitivity test to assess the effectiveness and stability of MR results.

All statistical analyses were performed using R (version 3.6.1) software with the R package “TwosampleMR.” Statistical significance was set at *P* < 0.05. Since publicly available summary data were used in this study, ethical approval was not needed.

## Results

### Selection of Instrumental Variables

In this study, significant (*P* < 5 × 10^–8^) and independent (*r*^2^ < 0.5) SNPs were extracted, weak IVs were excluded based on *F* statistics ≥ 10, and the remaining SNPs were selected for MR analysis. Finally, a total of 72 PD-related SNPs, 125 AD-related SNPs, and 12 IS-related SNPs were selected from GWAS summary data for MR analysis. These SNPs are listed in Supplementary File 1.

### Causal Relationship of Parkinson’s Disease and Osteoarthritis

**FIGURE 2:**
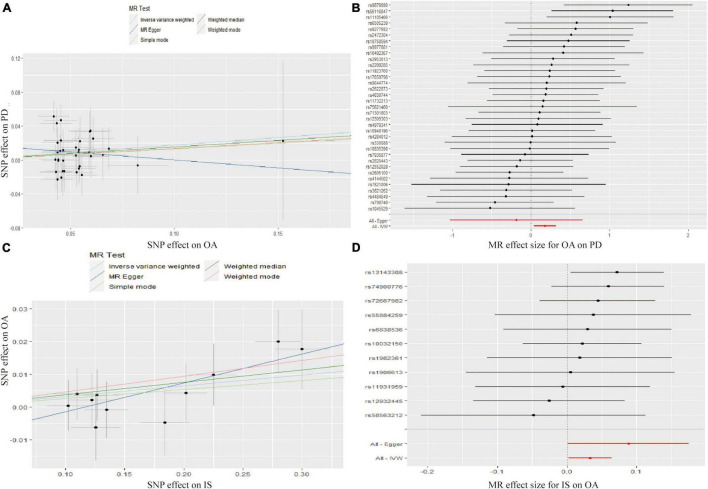
Scatter plots showed the causal effect of OA on PD **(A)**, IS on OA **(C)**. The slopes of line represent the causal effect of each method, respectively. Forrest plot of the causal effects of OA associated SNPs on PD **(B)**, IS associated SNPs on OA **(D)**. SNP, single nucleotide polymorphism; OA, osteoarthritis; PD, Parkinson’s disease; IS, Ischemic stroke; IVW, inverse variance weighted.

**FIGURE 3:**
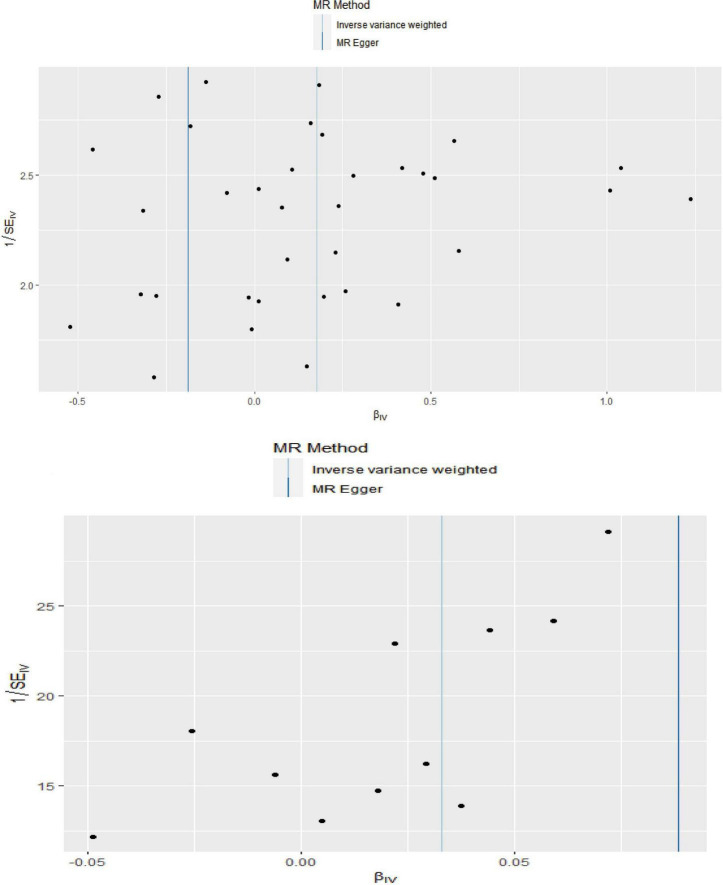
Funnel plot showed there were no significant heterogeneity among SNPs.

**FIGURE 4:**
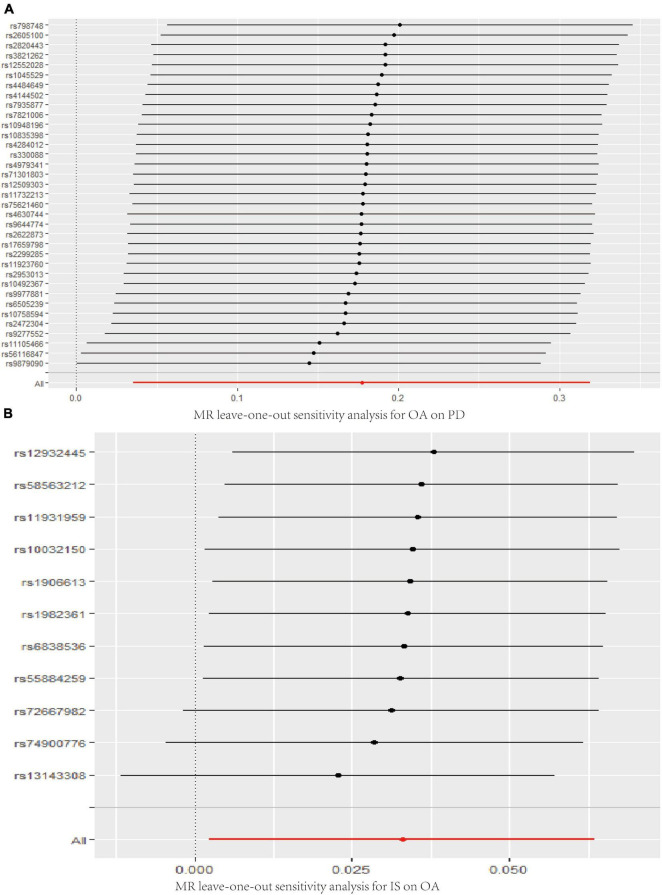
Leave-one-out analysis plots for OA on PD **(A)**, IS on OA **(B)**. MR, Mendelian randomization; OA, osteoarthritis; PD, Parkinson’s disease; IS, Ischemic stroke.

The MR analysis results with regard to the causal effect of OA on PD are listed in Table 1 and Figures 2A,B. The results are reported as odds ratios (ORs). As the results showed, the causal effect of OA on PD estimated by IVW suggested a positive effect (OR = 1.194, 95%CI = 1.036, 1.378; *P* = 0.0144). Cochran’s *Q* test showed that there was no heterogeneity (*Q* = 34; *P* = 0.595; Figure 3), and there was no directional pleiotropy (intercept = 0.0219; *P* = 0.395). Finally, to evaluate whether these results were affected by individual SNPs, the leave-one-out sensitivity test was performed, and the results showed that the causal effect of PD on OA did not significantly fluctuate with any single SNP leave-out (Figure 4A). Furthermore, the causal effect of PD on OA was assessed. The results are provided in Supplementary File 2. There was heterogeneity presence (*Q* = 132.48; *P* = 9.57 × 10^–6^) and pleiotropy absence (intercept = –4.554 × 10^–4^; *P* = 0.285) in the MR analysis results. According to Nazarzadeh et al. (2020), the result analyzed by weighted median was recommended to be adopted. The results showed that there was a null effect of PD on OA (OR = 0.980, 95%CI = 0.956, 1.004; *P* = 0.127). In summary, our results showed that OA had a positive effect on PD.

**TABLE 1:** The Mendelian randomization (MR) analysis results with regard to causal effect of OA on PD.

Exposure	Method	SNP (*n*)	OR	OR 95%CI	*P*-value
OA	MR Egger	35	0.828	0.356, 1.926	0.664
OA	Weighted median	35	1.167	0.961, 1.417	0.119
OA	Inverse variance weighted	35	1.194	1.036, 1.378	0.0144
OA	Simple mode	35	1.140	0.771, 1.685	0.515
OA	Weighted mode	35	1.146	0.771, 1.704	0.504

*OA, osteoarthritis; PD, Parkinson’s disease; SNP, single nucleotide polymorphism; OR, odds ratio; CI, confidence interval.*

### Causal Relationship of Alzheimer’s Disease and Osteoarthritis

The MR analysis results regarding the causal effect of AD on OA are listed in Supplementary File 2. Since there was heterogeneity (*Q* = 180.72; *P* = 3.478 × 10^–6^) and pleiotropy (intercept =–4.079 × 10^–3^; *P* = 0.0382), the results analyzed by MR-Egger were adopted. The results showed that there was a null effect of AD on OA (OR = 0.989, 95%CI = 0.977, 1.0007; *P* = 7.010 × 10^–2^; Supplementary File 2). The results were not affected by a single SNP (Supplementary Figure 1A). The MR analysis with regard to the causal effect of OA on AD also showed that there was a null effect of OA on AD (OR = 1.064, 95%CI = 0.928, 1.219; *P* = 0.374; Supplementary File 2). In total, we showed that there was no causal relationship between OA and AD.

### Causal Relationship of Ischemic Stroke and Osteoarthritis

Finally, the causal relationship between IS and OA was assessed by MR. The results are shown in Table 2 and Figures 2C,D. The MR analysis results by IVW suggested that IS had a positive effect on OA (OR = 1.033, 95%CI = 1.002, 1.066; *P* = 0.0355). There was no heterogeneity (*Q* = 4.475; *P* = 0.923; Figure 3) or pleiotropy (intercept =–0.0102; *P* = 0.214), and the MR analysis results were stable and were not significantly affected by individual SNPs (Figure 4B). Moreover, the causal effect of OA on IS was also assessed, the IVW results suggested that there was a null effect of OA on IS (OR = 0.983, 95%CI = 0.872, 1.087; *P* = 0.780), and the result was reliably tested by the leave-one-out sensitivity test (Supplementary Figure 1B). In summary, our results showed that there was a null effect of OA on IS, but a positive effect of IS on OA.

**TABLE 2:** The MR analysis results with regard to causal effect of IS on OA.

Exposure	Method	SNP (*n*)	OR	OR 95%CI	*P*-value
IS	MR Egger	11	1.092	1.002, 1.191	0.0769
IS	Weighted median	11	1.039	0.999, 1.081	0.0577
IS	Inverse variance weighted	11	1.033	1.002, 1.066	0.0355
IS	Simple mode	11	1.028	0.959, 1.100	0.455
IS	Weighted mode	11	1.049	0.993, 1.107	0.116

*OA, osteoarthritis; IS, ischemic stroke; SNP, single nucleotide polymorphism; OR, odds ratio; CI, confidence interval.*

## Discussion

Osteoarthritis is the main cause of pain and dysfunction in the elderly, and its incidence is increasing annually. Pathological changes include cartilage degeneration and osteophyte hyperplasia. In recent decades, the role of local inflammation in the development of OA has attracted increasing attention. Proinflammatory factors are important mediators of inflammatory reactions. The levels of proinflammatory factors, such as interleukin (IL), tumor necrosis factor (TNF), and matrix metalloproteinase (MMP), have been shown to be increased in OA, leading to systemic and local inflammatory reactions, accelerating the destruction of cartilage, and promoting the development of OA (Goldring and Otero, 2011; Kapoor et al., 2011). Parkinson’s disease is one of the most common neurodegenerative dyskinesia diseases in elderly people and is characterized by tremor, muscle rigidity, slow movement, and postural gait disorder. The pathological mechanism of PD is complex and yet to be fully elucidated. The role of inflammatory reactions in the pathogenesis of PD has aroused widespread concern. A growing number of studies have demonstrated that microglia-mediated inflammatory reactions are involved in the genesis and development of PD (Dorszewska et al., 2014; Qin et al., 2021). Thus, the development of anti-inflammatory drugs to delay or prevent the occurrence and development of PD has been the focus in recent years (Pajares et al., 2020). Inflammatory reactions play an indispensable role in the pathogenesis of both PD and OA. Many studies have focused on the causal relationship between PD and OA. Recently, Feng et al. (2021) suggested that patients with OA were at a higher risk of developing PD based on a population-based longitudinal follow-up study. Rugbjerg et al. suggested that prolonged non-steroidal anti-inflammatory drug (NSAID) treatment for osteoarthritis did not lower the risk of PD, while the slightly increased risk of PD might be attributed to the lower smoking prevalence in patients with OA (Roper et al., 2020). Consistent with Feng et al. (2021), this study showed that OA had a positive causal effect (OR = 1.194, 95%CI = 1.036, 1.378; *P* = 0.0144) on PD, and this result was stable and reliable. Based on the above results, we can conclude that OA might promote the development of PD; thus, as for OA patients, the prevention strategies of PD should also be adopted. For example, physical activity should be recommended for patients with OA, since it has been reported that physical activity could decrease the risk of PD. Moreover, when initial symptoms of PD, such as depression, occur in OA patients, they should be determined in a timely manner, and both physical and medical treatment should be administrated (Jacob et al., 2021).

Alzheimer’s disease is a neurodegenerative disease. Previous studies have shown that inflammatory reactions, such as IL-1β, IL-1L, and TNF-α, play an important role in the pathogenesis of AD (Akiyama, 2000; Holmes, 2013). A growing number of studies have suggested that OA may promote the development of AD in both basic and clinical research (Kyrkanides et al., 2011; Huang et al., 2015). In addition to inflammatory reactions, pain is another characteristic that may be caused by inflammation, nerve remodeling, etc. Pharm et al. showed that pain interference with normal activities in the absence of OA was associated with AD and related dementias (Ikram et al., 2019). However, in this study, there was no evidence suggesting a causal effect of OA on AD (OR = 1.064, 95%CI = 0.928, 1.219; *P* = 0.374), nor the causal effect of AD on OA (OR = 0.989, 95%CI = 0.977, 1.0007; *P* = 7.010 × 10^–2^). These results suggest that OA may not be related to the risk of AD. This might be attributed to the different populations enrolled in different studies; thus, more studies should be performed to elucidate the causal relationship between OA and PD.

Ischemic stroke is a cerebrovascular disease with an increasing incidence, has a high disability and mortality rate, and leads to sequelae or serious complications, significantly affecting the quality of life. The relationship between stroke and OA has been repeatedly reported (Hsu et al., 2017; Yeh et al., 2021). For example, Hsu et al. (2017) performed a population-based cohort study and concluded that OA might contribute to the increased risk of stroke. In this study, our MR analysis showed that there was no evidence of a causal effect of OA on IS (OR = 0.983, 95%CI = 0.872, 1.087; *P* = 0.780). This is different from some previous studies (Jacob et al., 2021; Yeh et al., 2021), suggesting that OA might not increase the risk of IS, but the treatment of OA might. Conversely, our results supported the positive causal effect of IS on OA (OR = 1.033, 95%CI = 1.002, 1.066; *P* = 0.0355). These results should be interpreted cautiously, as there is insufficient evidence regarding the risk of OS in patients with IS.

This was the first study to evaluate the causal relationship between CNSD and OA using two-sample MR analysis, which was free of biases and confounders, and reverse causality could also be avoided. However, there are also some limitations to this study: (1) the MR analysis was based on European ancestry, and these results might be different with the change of ancestry; (2) this study focused on the causal relationship between CNSD and OA, but whether the treatment of CNSD has a causal association with OA remains unclear.

## Conclusion

This study showed a positive causal effect of OA on PD and IS on OA, which might deepen our understanding of these senile diseases, so as to provide guidance on prevention and treatment.

## Data Availability Statement

The original contributions presented in the study are included in the article/Supplementary Material, further inquiries can be directed to the corresponding author.

## Author Contributions

YC, GZ, and JL performed the study and wrote the manuscript. RZ and LL revised the manuscript. XD designed the study. All authors contributed to the article and approved the submitted version.

## Conflict of Interest

The authors declare that the research was conducted in the absence of any commercial or financial relationships that could be construed as a potential conflict of interest.

## Publisher’s Note

All claims expressed in this article are solely those of the authors and do not necessarily represent those of their affiliated organizations, or those of the publisher, the editors and the reviewers. Any product that may be evaluated in this article, or claim that may be made by its manufacturer, is not guaranteed or endorsed by the publisher.

## Footnotes

1
https://gwas.mrcieu.ac.uk/datasets/ieu-b-7/

## Supplementary Material

Supplementary Figure 1 Leave-one-out analysis plots for AD on OA **(A)** and OA on IS **(B)**. MR, Mendelian randomization; OA, osteoarthritis; AD, Alzheimer’s disease; IS, ischemic stroke.
