# Supplementary figures and images for: Causal Relationships Between Osteoarthritis and Senile Central Nerve System Dysfunction: A Bidirectional Two-Sample Mendelian Randomization Study
Source: Front Aging Neurosci. 2022 Mar 4;13:793023. doi: 10.3389/fnagi.2021.793023 (PMC8934417; doi:10.3389/fnagi.2021.793023)

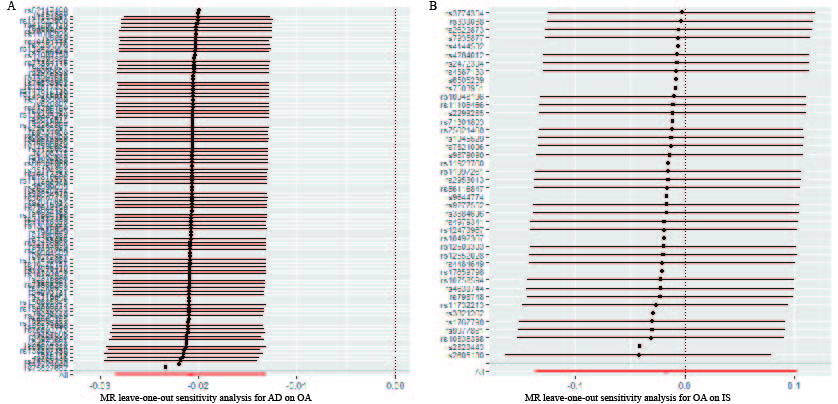

Supplement: Supplementary Figure 1 — Leave-one-out analysis plots for AD on OA (A) and OA on IS (B). MR, Mendelian randomization; OA, osteoarthritis; AD, Alzheimer’s disease; IS, ischemic stroke. [file Image_1.JPEG]
