# Supplementary material for: Causal Relationships Between Osteoarthritis and Senile Central Nerve System Dysfunction: A Bidirectional Two-Sample Mendelian Randomization Study
Source: Front Aging Neurosci. 2022 Mar 4;13:793023. doi: 10.3389/fnagi.2021.793023 (PMC8934417; doi:10.3389/fnagi.2021.793023)
Supplement: Supplementary file 2 [file Data_Sheet_1.docx]

**Supplementary Table 1.** The MR analysis results with regard to causal effect of PD on OA

| **Exposure** | **Method** | **SNP (n)** | **OR** | **OR 95%CI** | ***P*-value** |
| --- | --- | --- | --- | --- | --- |
| PD | MR Egger | 71 | 0.985 | 0.933, 1.041 | 0.603 |
| PD | Weighted median | 71 | 0.980 | 0.956, 1.004 | 0.127 |
| PD | Inverse variance weighted | 71 | 0.958 | 0.939, 0.977 | 2.26×10^-5^ |
| PD | Simple mode | 71 | 1.0001 | 0.946, 1.059 | 0.982 |
| PD | Weighted mode | 71 | 0.999 | 0.951, 1.050 | 0.985 |

PD: Parkinson's disease; OA: osteoarthritis; SNP: single nucleotide polymorphism; OR: odds ratio; CI: Confidence interval.

**Supplementary Table 2.** The MR analysis results with regard to causal effect of AD on OA

| **Exposure** | **Method** | **SNP (n)** | **OR** | **OR 95%CI** | ***P*-value** |
| --- | --- | --- | --- | --- | --- |
| AD | MR Egger | 104 | 0.989 | 0.977, 1.0007 | 7.010×10^-2^ |
| AD | Weighted median | 104 | 0.987 | 0.977, 0.997 | 1.336×10^-2^ |
| AD | Inverse variance weighted | 104 | 0.979 | 0.972, 0.987 | 1.190×10^-7^ |
| AD | Simple mode | 104 | 0.956 | 0.934, 0.979 | 3.653×10^-4^ |
| AD | Weighted mode | 104 | 0.988 | 0.979, 0.997 | 7.823×10^-3^ |

AD: Alzheimer's disease; OA: osteoarthritis; SNP: single nucleotide polymorphism; OR: odds ratio; CI: Confidence interval.

**Supplementary Table 3.** The MR analysis results with regard to causal effect of OA on PD

| **Exposure** | **Method** | **SNP (n)** | **OR** | **OR 95%CI** | ***P*-value** |
| --- | --- | --- | --- | --- | --- |
| OA | MR Egger | 32 | 2.497 | 0.897, 6.950 | 0.0899 |
| OA | Weighted median | 32 | 1.058 | 0.888, 1.262 | 0.527 |
| OA | Inverse variance weighted | 32 | 1.064 | 0.928, 1.219 | 0.374 |
| OA | Simple mode | 32 | 0.808 | 0.542, 1.206 | 0.305 |
| OA | Weighted mode | 32 | 0.835 | 0.552, 1.262 | 0.398 |

AD: Alzheimer's disease; OA: osteoarthritis; SNP: single nucleotide polymorphism; OR: odds ratio; CI: Confidence interval.

**Supplementary Table 4.** The MR analysis results with regard to causal effect of OA on IS

| **Exposure** | **Method** | **SNP (n)** | **OR** | **OR 95%CI** | ***P*-value** |
| --- | --- | --- | --- | --- | --- |
| OA | MR Egger | 42 | 0.994 | 0.448, 2.204 | 0.988 |
| OA | Weighted median | 42 | 0.976 | 0.824, 1.156 | 0.782 |
| OA | Inverse variance weighted | 42 | 0.983 | 0.872, 1.087 | 0.780 |
| OA | Simple mode | 42 | 0.992 | 0.696, 1.416 | 0.967 |
| OA | Weighted mode | 42 | 1.019 | 0.710, 1.464 | 0.916 |

IS: ischemic stroke; OA: osteoarthritis; SNP: single nucleotide polymorphism; OR: odds ratio; CI: Confidence interval.
